# Pea Peptide Supplementation in Conjunction With Resistance Exercise Promotes Gains in Muscle Mass and Strength

**DOI:** 10.3389/fnut.2022.878229

**Published:** 2022-07-07

**Authors:** Shaohui Jia, Qiming Wu, Shue Wang, Juntao Kan, Zhao Zhang, Xiping Zhang, Xuejun Zhang, Jie Li, Wenhan Xu, Jun Du, Wei Wei

**Affiliations:** ^1^Hubei Collaborative Innovation Center for Sports Intervention and Health Promotion, Wuhan Sports University, Wuhan, China; ^2^Amway (Shanghai) Innovation & Science Co., Ltd., Shanghai, China; ^3^School of Public Health, Shandong University, Jinan, China; ^4^Zhong Shi Du Qing (Shandong) Biotechnology Company, Heze, China; ^5^College of Food Science and Engineering, Northwest A&F University, Yangling, China

**Keywords:** pea peptide, resistance exercise, muscle mass, muscle strength, muscle growth

## Abstract

It is generally considered that protein supplementation and resistance exercise significantly increase muscle mass and muscle growth. As the hydrolysis products of proteins, peptides may play the crucial role on muscle growth. In this study, male rats were orally administrated 0.4 g/kg body weight of pea peptide combined with 8 weeks of moderate intensity resistance exercise training. After treatment, the body gains, upper limb grip, muscle thickness, and wet weight of biceps brachii were tested, and the cross-sectional area of biceps brachii muscle fiber and the types of muscle fibers were determined by HE staining, immunofluorescence staining, and lactate dehydrogenase activity, respectively. Western blot analysis was used to investigate the level of growth-signaling pathway-related proteins. The results showed that pea peptide supplementation combined with resistance exercise training significantly increased body weight, upper limb grip, muscle thickness, wet weight of biceps brachii, and cross-sectional area of muscle fiber. Meanwhile, pea peptide supplementation obviously elevated the ratio of fast-twitch fiber (type II) and the expression of muscle growth-signaling pathway-related proteins. In addition, the PP2 oligopeptide in pea peptide with the amino acid sequence of LDLPVL induced a more significant promotion on C2C12 cell growth than other oligopeptides.

## Introduction

Skeletal muscle mass is an integral body tissue playing the key roles in strength, performance, and metabolic regulation. Skeletal muscle mass is the product of continuous and simultaneous processes of muscle protein synthesis (MPS) and muscle protein breakdown (MPB) and the net balance between these two processes determines whether muscle mass increases, decreases, or remains constant (1).

It is well known that protein supplementation including animal protein such as meat, fish, dairy products, eggs, and plant protein such as tofu, legumes, and quinoa is necessary for muscle growth in responsible to resistance exercise. A general view within the sport nutrition community is that animal proteins and whey protein, in particular, are more effective in building muscle in response to resistance exercise training (2). Whereas some researchers regarded that plant proteins such as soy protein may have a number of advantages over animal protein including lowering blood cholesterol levels (3), other researchers also suggested that any nutritional supplement that could increase protein accretion in muscle would maximize resistance training effects by enhancing muscle anabolism (4). At the same time, studies have also shown that there are no differences not only between plant protein and whey protein, but also between soy protein and general animal protein when it comes to building muscle (5). Soy protein is often considered to be the quintessential plant protein and plays a positive role on increasing muscle mass (6). Pea protein is another important plant protein, and one study has confirmed that pea protein supplementation induced a greater increase on muscle thickness as compared to placebo (7).

It is reported that the intake of essential amino acids (EAAs) such as leucine, isoleucine, lysine, methionine, phenylalanine, threonine, tryptophan, and valine after physical exercise can ensure the optimal protein synthesis, and the net protein synthesis increases when EAAs are consumed before and after resistance exercise (8, 9). In the recent years, oligopeptides have attracted more and more attention in many fields owing to their high biological activity and absorbability (10–12). Unfortunately, more studies are still needed to determine the role of oligopeptides in promoting muscle growth.

Despite supplementation with plant derived proteins has been confirmed to promote muscle growth in response to resistance exercise training, few studies have focused on the effect of pea peptides on skeletal muscle growth. Therefore, in this study, pea oligopeptide (PP) was first obtained by enzymatic hydrolysis of pea protein. Then, 2-month-old rats were orally administrated pea oligopeptide and performed 8 weeks of resistance exercise training to evaluate the effect of pea oligopeptide supplementation combined with resistance exercise on skeletal muscle growth and explore new functional substances for combating muscle atrophy.

## Materials and Methods

### Pea Oligopeptide Preparation

The pea protein was diluted to 7.5% solution with free ionic water and rise the temperature to 55°C. Then, the solution was adjusted to pH 8.5 with 1 mol/L NaOH and added into 1.2% alkaline protease to hydrolyze the proteins for 6 h. After treatment, the enzyme was inactivated at 100°C for 20 min. Subsequently, the hydrolysate was filtered through a regenerated cellulose dialysis membrane with a molecular weight of 1,000 Dalton. Finally, the filtrate was collected and freeze-dried to obtain pea oligopeptide.

### Determination of Amino Acid Composition of Pea Peptide

The above-obtained sample was further purified by a high-performance liquid chromatograph (HPLC, Agilent 1100, Tokyo, Japan), and then, the purified products of RP-HPLC were collected to determine the amino acid composition of pea peptide using Hitachi Amino Acid Analyzer (LA8900, Hitachi, Japan).

### Separation of Pea Peptides by HPLC

The above peptides were further separated and purified by reverse HPLC under the following conditions: the mobile phase consisted of 95% acetonitrile and 5% water containing 0.1% trifluoroacetic, and the flow rate was 1.0 ml/min. After separation, the peak tip solution was collected to determine the amino acid sequence.

### Animals Grouping and Treatment

A total of 30 male Sprague-Dawley (SD) rats at the age of 8 weeks were obtained from Hubei Provincial Center for Disease Control and Prevention (license no. scxk (E) 2011-0012). All animals were first acclimated for at least 1 week and then kept under the following conditions: specific pathogen free, 12-h light–dark cycles at 22–24°C, and free access to food and water except when fasting tests were required. All experiments were conducted under the institutional guidelines of the Institutional Animal Care and Use Committee at Wuhan Sports University for the humane treatment of laboratory animals. Finally, the rats were randomly divided into control group (CN), resistance exercise training group (RT), and pea peptide combined with resistance training group (PP+RT), and 10 rats were fed in each group. The rats in RT group were carried out 8 weeks of resistance exercise training, the rats in PP + RT group were daily intragastrically administered with 0.4 g/kg body weight of pea peptides and then performed resistance exercise training for 8 weeks, and the rats in CN group were daily intragastrically administered with same volume of saline. The detailed exercise protocol is as follows: after a week of familiarization with the ladder, the rats of RT and PP +RT group were performed 8 weeks of ladder climbing exercise on a 1-m ladder with a 2-cm grid ladder inclined at 85° with weights attached to their tails. The maximum load carried successfully throughout the length of the ladder was considered to be the maximum load capacity for the training session, and the maximum load capacity was detected at weeks 1, 2, 4, 6, and 8. Each rat carried a load that was 50% of its body mass at the first training week and exercise load added to 100% of its body weight until the end of the 8th week with the frequency of three times per week, and 60 s of rest interval between repetitions and 5 min of rest interval between sets.

### HE Staining

Biceps brachii muscle tissues of rats were harvested from the rats and fixed with 4% paraformaldehyde (PFA) overnight. The tissues embedded in paraffin were sectioned into 4 μm thickness, followed by deparaffinization and rehydration, and finally stained with hematoxylin and eosin following the manufacturer's introductions (Solarbio, Beijing, G1121).

### Cell Culture and Treatment

C2C12 cell line was purchased from China Center for Type Culture Collection (CCTCC) and cultured with DMEM (Gibco, C11995500BT) containing 10% fetal bovine serum, 100 U/ml penicillin, and 100 mg/ml streptomycin in six well plates at 37°C. When the cells grew to 70% of the culture plate, 0.1 mg/ml of pea peptide components 1, 2, 3, 4, and 5 prepared by HPLC was added and incubated for 48 h. After treatment, the cells were harvested to perform western blot analysis.

### Immunofluorescence Staining

Biceps brachii muscle tissues of rats were fixed with 4% PFA (Solarbio, Beijing, P1110) and embedded in paraffin. The 4-μm sections were prepared, followed by deparaffinization, rehydration, and quenching endogenous peroxidases with 0.3% H_2_O_2_ (in PBS) for 10 min. Then, the tissue sections were incubated with antigen retrieval buffer (Solarbio, C1032) at 95°C for 20 min, permeabilized by 0.1% Triton X-100, and incubated with anti-fast skeletal myosin heavy chain antibody (MYH1, Abcam, 1:100, ab133567) and anti-slow skeletal myosin heavy chain antibody (MYH7, Abcam, 1: 500, ab234431) overnight at 4°C, followed by incubating with a secondary antibody goat anti-rat IgG H&L with FITC label (Alexa Fluor® 488) and goat anti-rabbit IgG H&L with CY3 label (Alexa Fluor® 647) for 60 min. After treatment, micromorphology was detected by Fluorescent Microscopy (IX83, Olympus). FITC glows green by excitation wavelength 465–495 nm and emission wavelength 515–555 nm; CY3 glows red by excitation wavelength 510–560 nm and emission wavelength 590 nm.

### Determination of Lactate Dehydrogenase Activity

The lactate dehydrogenase activity of skeletal muscle was determined by enzyme-linked immunosorbent assay (ELISA) analysis. Briefly, 0.1 g of biceps brachii muscle tissues was cut and added into 1 ml of extracting solution, then, the tissues were homogenized for 3 min at 65 Hz in a pre-cooled homogenizer at 4°C, and the homogenate was centrifuged at 4°C for 10 min with the speed of 8,000 *g*. After treatment, the supernatant was collected to detect the activity of lactate dehydrogenase according to the instructions (Solarbio, bc0685).

### Western Blot Analysis

Muscle tissue or cell lines were put into the pre-cooled saline solution to remove blood and connective tissues. After the tissues were cut, the cell lysis buffer and protease inhibitor (PMSF) were added into the centrifuged tube according to the ratio of 100:1 and then homogenized for 5 min at 65 Hz in a pre-cooled homogenizer at 4°C. The homogenized samples were treated with ultrasonic for 5 min and centrifuged at 4°C for 10 min, and then, the supernatant was collected to measure the protein concentration using BCA kit (Beyotime Institute of Biotechnology, China). The prepared total protein was separated by sodium dodecyl sulfate–polyacrylamide gel electrophoresis (SDS-PAGE) at 80 V and transferred onto a PVDF membrane for 2.5 h at 350 mA. The PVDF membrane was blocked for 2 h at room temperature with 5% nonfat milk and then washed three times with Tris Buffered Saline with 0.5% of Tween (TBST). Subsequently, the membrane was incubated with primary antibody including IGF-1R, myostatin, CDK6, cyclin D1, t-AMPK, and p-AMPK (Cell Signaling Technology, Danvers, MA, USA) overnight at 4°C, respectively, and the primary antibody GAPDH (all from Cell Signaling Technology, Danvers, MA, USA) was used as the reference antibodies, respectively. Following incubation, the membrane was washed with TBST and incubated with secondary antibody (Abcam, UK) for 1 h at room temperature. Finally, the membranes were developed using an enhanced chemiluminescence (ECL) reagent (Pierce Biotechnology, Rock-ford, IL, USA). All blots were quantified using ImageJ software (National Institutes of Health, USA).

### Cell Proliferation Assay

Cell proliferation was evaluated by MTT assay. C2C12 cells were seeded in a 96-well cell culture plate at a density of 1 × 10^4^ cells/well and incubated with pea peptide (10 μg/ml) for 12, 24, or 48 h. Untreated cells served as the control group. After treatment, MTT was added into each well at a final concentration of 5 mg/ml, and the cells were further incubated at 37°C for 4 h. Dimethyl sulfoxide (100 μl) was added into each well after removing the medium. After shaking the plates for 5 min, the absorbance of the mixture was measured at 490 nm using a microplate ELISA reader (SpecterMax i3x, Austria).

### Statistical Analysis

All values were performed using GraphPad prism 6.0 (GraphPad Software, Inc. San Diego, CA, USA) software. Data were presented as mean ± standard deviation (M ± SD). Statistical analysis was performed using one-way ANOVA between groups. Tukey's *post hoc* test was performed to express the mean difference between groups. The statistically significant difference was considered at *p* < 0.05.

## Results

### The Amino Acid Composition of Pea Peptide

The amino acid content of pea peptide is up to 93.08%. The content of Ser was 13.20%, which is the most abundant amino acids in pea peptide. Moreover, pea peptide is also rich in the branched chain amino acids (Val, Ile, and Leu) and account for 19.46%. In addition, pea peptide contains abundant hydrophobic amino acids, including Ala, Val, Leu, Gly, Phe, and Met, accounting for about 28.72% (Table 1).

**Table 1:** Amino acid composition of pea peptide (g / 100 g peptide).

**Retention time**	**Amino acid**	**Area**	**Percentage (%)**
4.733	Asp	5332554	12.12
5.380	Thr	1700427	3.67
5.973	Glu	2601175	7.02
6.673	Ser	7574282	13.20
7.293	Pro	783389	4.77
9.513	Gly	2478635	3.94
10.280	Ala	1866356	4.07
11.667	Cys	155165	0.59
12.273	Val	2869568	5.93
13.500	Met	436951	0.94
15.800	Ile	1926054	4.28
16.987	Leu	3430418	9.25
18.300	Tyr	646207	2.46
19.507	Phe	1746753	4.59
22.000	Lys	3693258	7.06
24.200	His	879921	2.00
28.240	Arg	2723907	7.19

### Pea Peptide Supplementation Combined With Resistance Exercise Induced Elevated Muscle Thickness and Body Weight Gains

**Figure 1:**
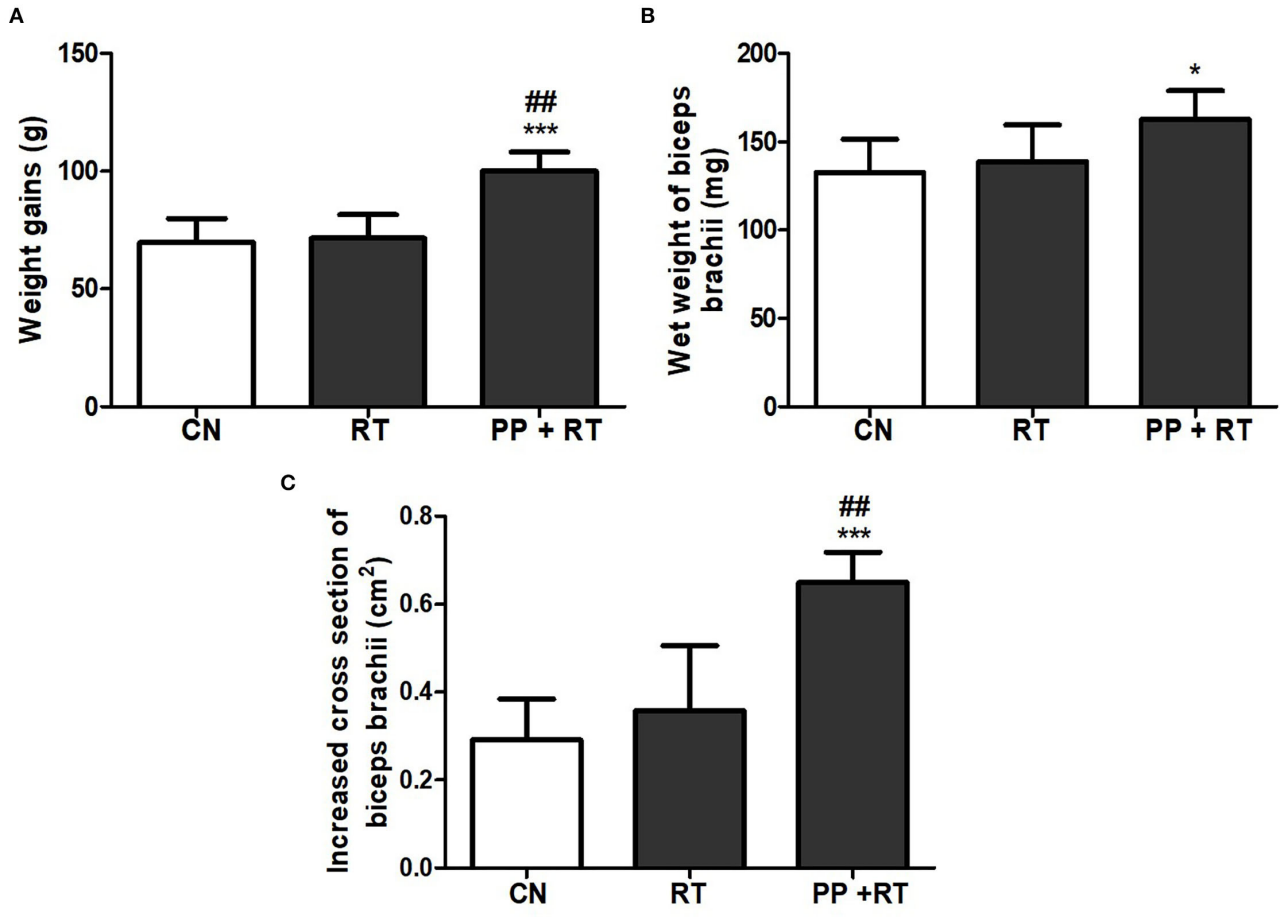
Pea peptide supplementation combined with resistance exercise induced elevated muscle thickness and weight gains. **(A)** Body weight gains of the rats in CN, RT, and PP+RT group. **(B)** Wet weight of biceps brachii in the rats of CN, RT, and PP+RT group. **(C)** Increased cross-section of biceps brachii in the rats of CN, RT, and PP+RT group. CN: Control group, the rats were intragastrically administered with same volume of saline; RT: Resistance training group, the rats were carried out 3 times each week of resistance exercise training; PP+RT: Pea peptide supplementation combined with resistance exercise training group, the rats were daily intragastrically administered with 0.4 g/kg body weight of pea peptides and performed three times each week of resistance exercise training. The significant difference between groups was validated with one-way ANOVA using GraphPad Prism 6, *p < 0.05, ***p < 0.01 vs. CN group, and ^##^*p* < 0.01 vs. RT group (*n* = 5/group). Data are presented as mean ± standard deviation (M ± SD).

As shown in Figure 1, compared with control and resistance training, treatment with pea peptide supplementation combined with resistance training resulted in an obvious increase on the body weight gains and the cross-section of biceps brachii (Figures 1A,C). We also observed that resistance exercise failed to enhance the wet weight of biceps brachii, whereas pea peptide combined with resistance exercise induced a significant increase on the wet weight of biceps brachii (Figure 1B).

### Pea Peptide Supplementation Combined With Resistance Exercise Increased Forelimb Grip and Muscle Cross-Section

**Figure 2:**
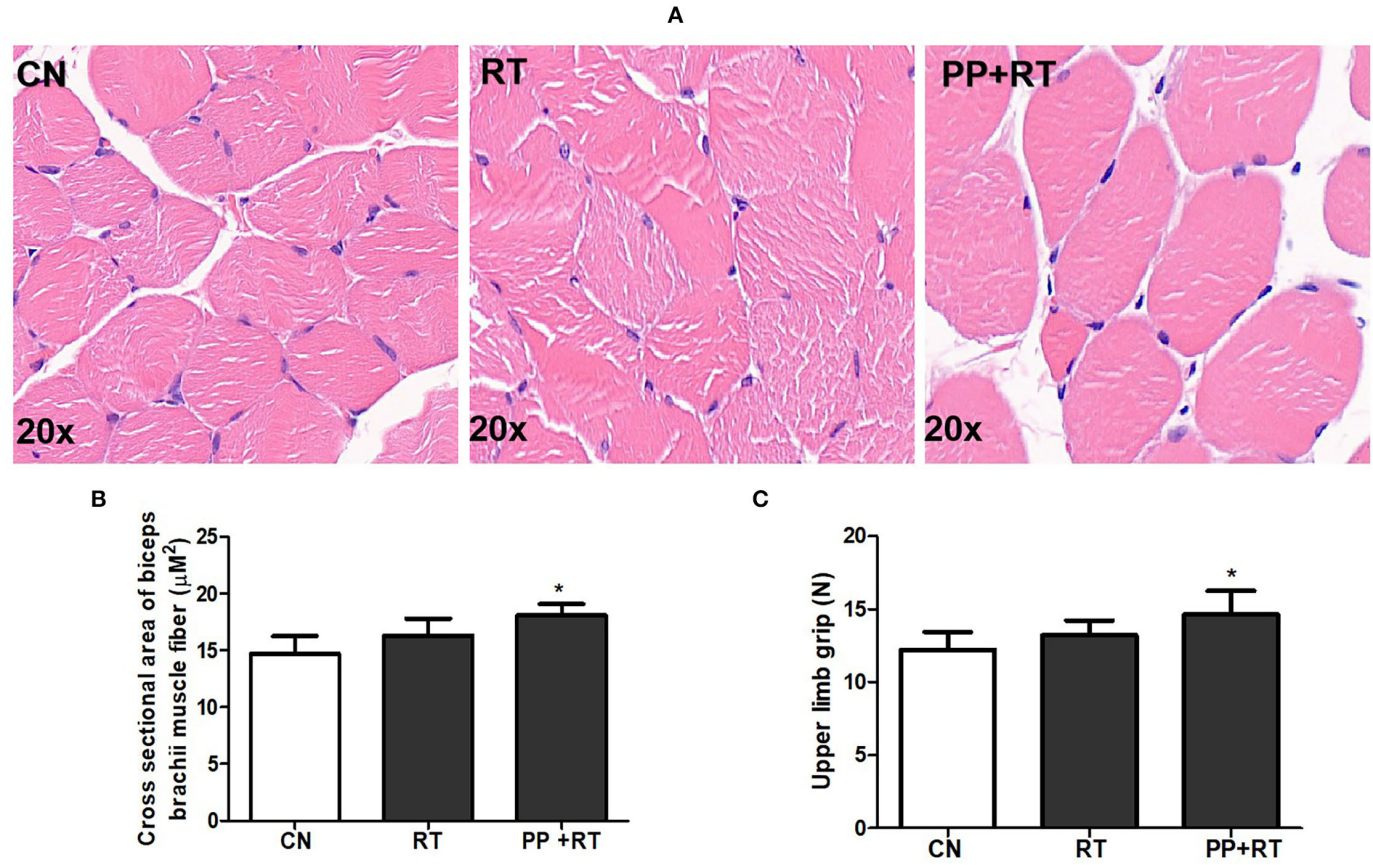
Pea peptide supplementation combined with resistance exercise increased cross-section of muscle fiber and forelimb grip in rats. **(A)** HE staining of biceps brachii in the rats of CN, RT, and PP+RT group (20 x); **(B)** Statistical analysis of **(A)**; **(C)** Upper limb grip of rats in CN, RT and PP+RT group; CN, Control group; RT, Resistance training group; PP+RT: Pea peptide supplementation combined with resistance training. The significant difference between groups was validated with one-way ANOVA using GraphPad Prism 6, *p < 0.05 vs. CN group (n=5/group). Data are presented as mean ± standard deviation (M ± SD).

We subsequently investigated the effect of pea peptide supplementation on muscle strength, and the results showed that the cross-sectional area of biceps brachii muscle fiber in PP+RT group was significantly higher than that in CN and RT group (Figures 2A,B) (*p* < 0.05). Correspondingly, the upper limb grip also presented an obvious elevation in the rats of PP+RT group compared to CN and RT group (Figure 2C) (*p* < 0.05).

### Pea Peptide Supplementation Combined With Resistance Exercise Enhanced the Ration of Type 2 Muscle Fiber

**Figure 3:**
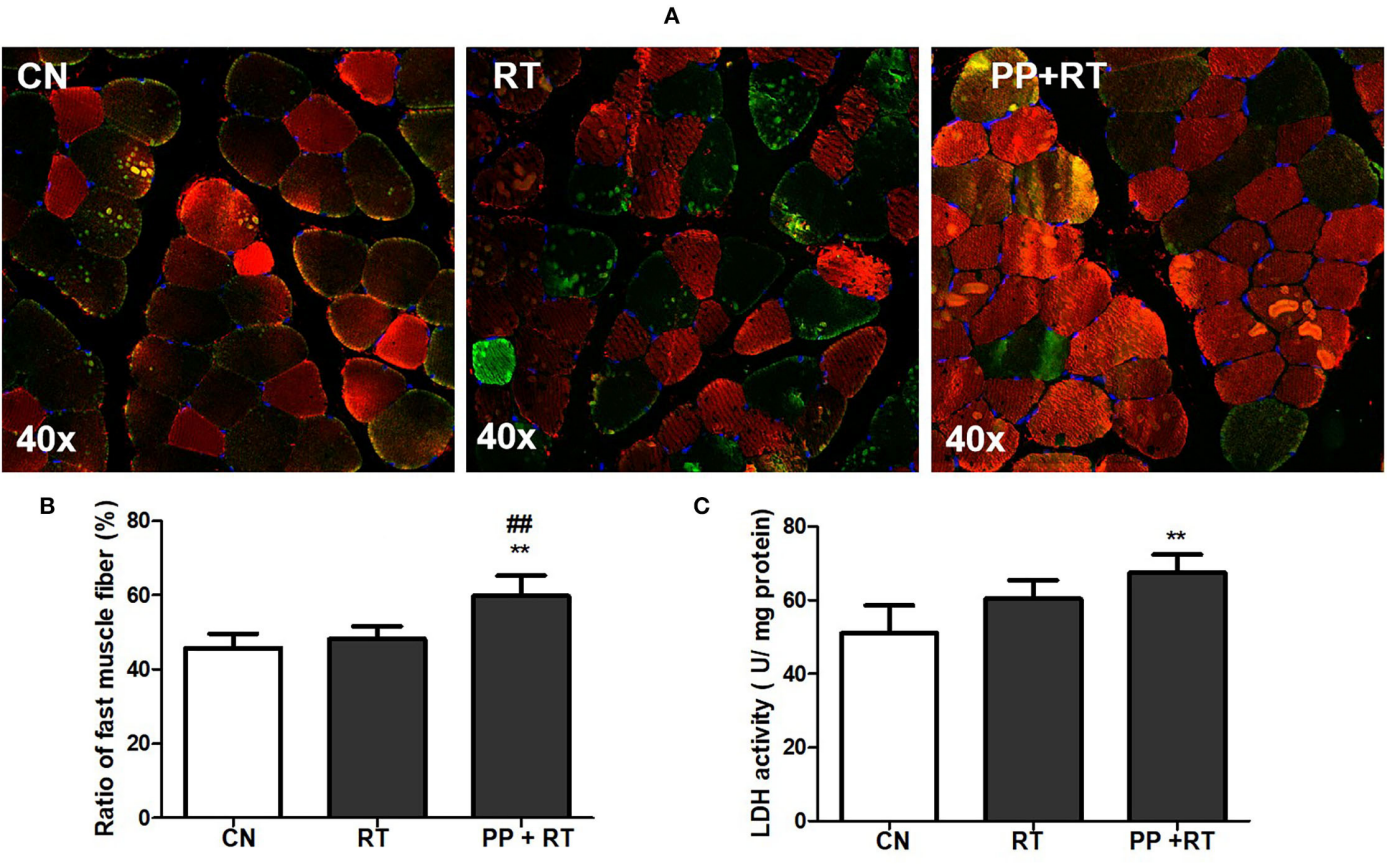
Pea peptide supplementation enhanced the proportion of type 2 muscle fiber. **(A)** Immunofluorescence staining of biceps brachii in the rats of CN, RT, PP+RT group (40 x). Fast muscle fibers were labeled with red fluorescence and slow muscle fibers were labeled with green fluorescence; **(B)** statistical analysis of **(A)**; **(C)** upper limb grip of rats in CN, RT, PP+RT group. CN: Control group; RT: resistance training group; PP+RT: pea peptide supplementation combined with resistance training. The significant difference between groups was validated with one-way ANOVA using GraphPad Prism 6, **p < 0.01 vs. CN group, and ^##^*p* < 0.01 vs. RT group (*n* = 5/group). Data are presented as mean ± standard deviation (M ± SD).

Immunofluorescence staining indicated that resistance exercise alone failed to alter the ratio of fast-twitch muscular fiber in biceps brachii muscle. Whereas pea peptide supplementation combined with resistance exercise induced a great increase on the ratio of fast-twitch muscular fiber (Figures 3A,B), meanwhile, the LDH activity of biceps brachii muscle in PP + RT group showed a significant elevation compared with CN and RT group (Figure 3C).

### Pea Peptide Supplementation Combined With Resistance Exercise Activated Muscle Growth-Signaling Pathway

**Figure 4:**
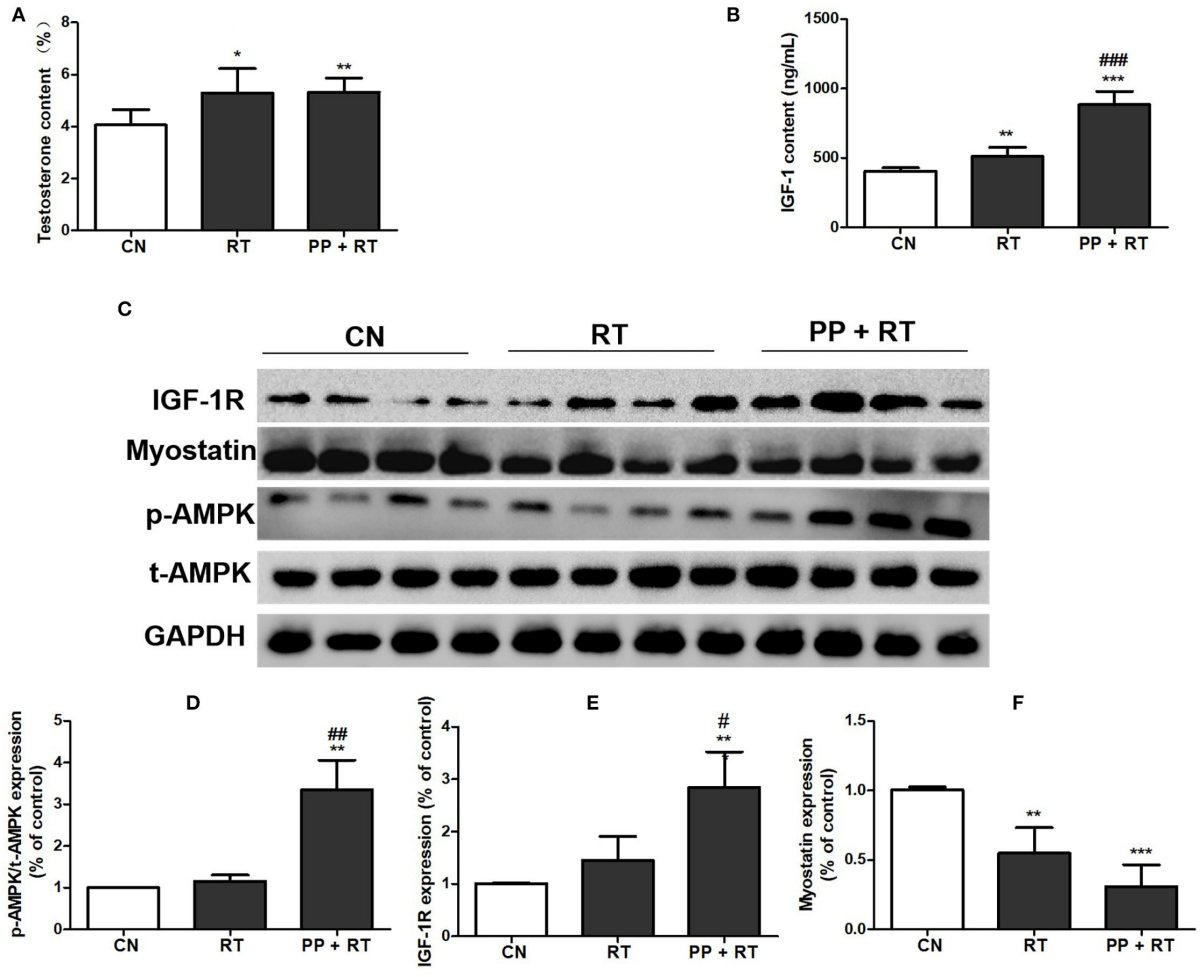
Pea peptide supplementation activated muscle growth-signaling pathway. **(A)** Circulating testosterone content of the rats in CN, RT, and PP + RT group; **(B)** circulating IGF-1 content of the rats in CN, RT, and PP + RT group; **(C)** p-AMPK, t-AMPK, IGF-1R, and myostatin expression of skeletal muscle detected by western blots analysis; **(D)** statistical analysis of p-AMPK expression; **(E)** statistical analysis of IGF-1R expression; **(F)** statistical analysis of myostatin expression. CN: Control group; RT: resistance training group; PP+RT: pea peptide supplementation combined with resistance training. The significant difference between groups was validated with one-way ANOVA using GraphPad Prism 6, *p < 0.05, **p < 0.01, ***p < 0.001 vs. CN group, and ^#^*p* < 0.05, ^##^*p* < 0.01, ^###^*p* < 0.001 vs. RT group (*n* = 5/group). Data are presented as mean ± standard deviation (M ± SD).

A significant improvement in muscle mass and muscle strength induced by pea peptide supplementation has been observed in this study, we subsequently investigated the effect of pea peptide supplementation on muscle growth-signaling pathway. The results showed that testosterone content of the rats in RT and PP+RT group was significantly higher than that in CN group (Figure 4A). We also observed a significant elevation on the concentration of circulating IGF-1 in RT group compared with CN group and a more significant increase on the concentration of circulating IGF-1 in PP+RT group compared with CN and RT group (Figure 4B). Western blot analysis also indicated that pea peptide supplementation combined with resistance exercise significantly enhanced the expression of IGF-1R and p-AMPK, while obviously reduced myostatin expression in skeletal muscle (Figures 4C–F).

### Component 5 of Pea Peptide Significantly Activated Muscle Growth-Signaling Pathway

**Figure 5:**
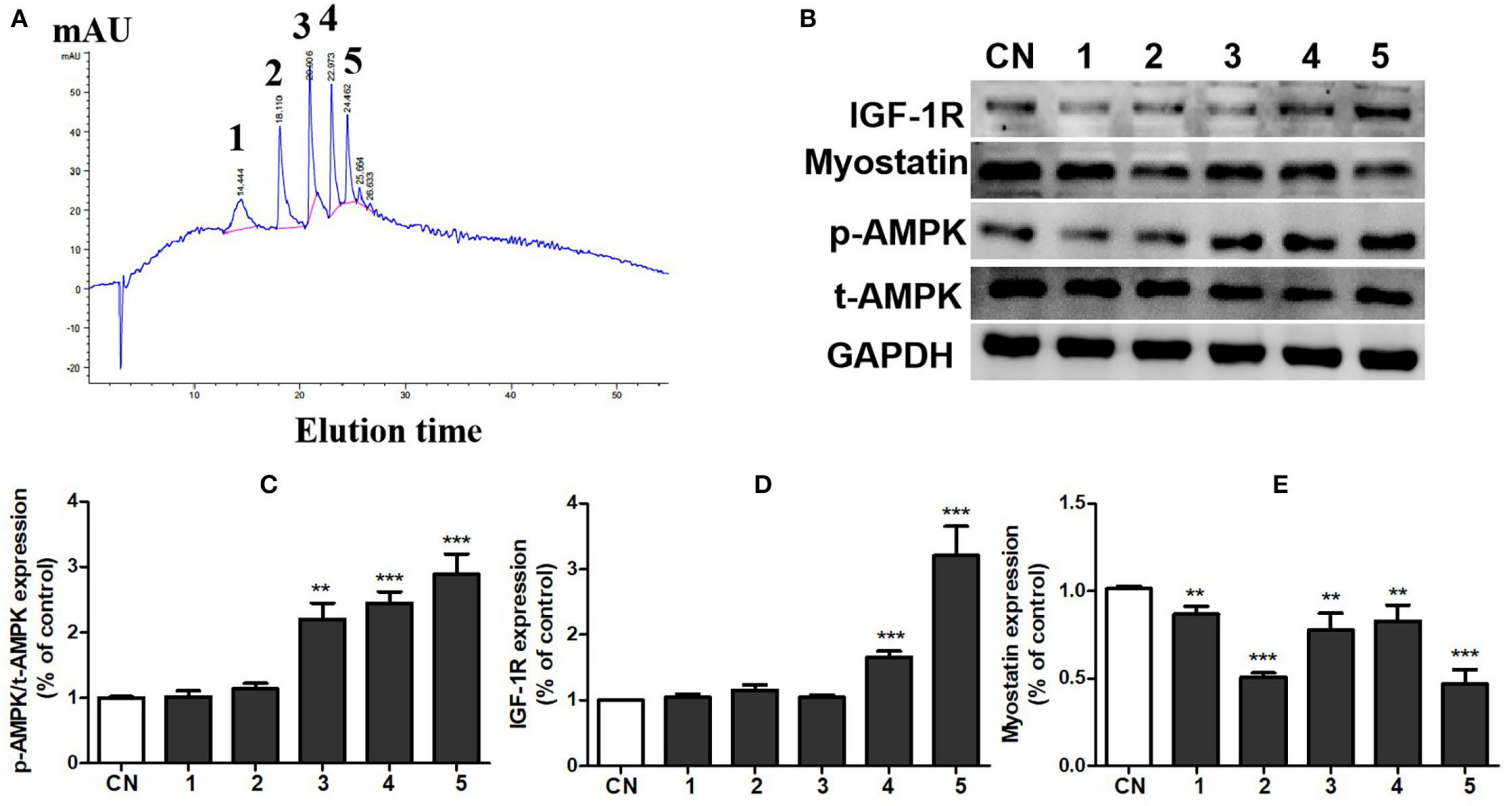
Component 5 of pea peptide significantly activated muscle growth-signaling pathway. **(A)** Separation of pea peptides by HPLC; **(B)** expression of t-AMPK, p-AMPK, myostatin, and IGF-1R investigated by western blots; **(C)** statistical analysis of p-AMPK and t-AMPK expression; **(D)** statistical analysis of IGF-1R expression; **(E)** statistical analysis of myostatin expression. CN: Control group; RT: resistance training group; PP+RT: pea peptide supplementation combined with resistance training. The significant difference between groups was validated with one-way ANOVA using GraphPad Prism 6, **p < 0.01, ***p < 0.001 vs. CN group (n = 5/group). Data are presented as mean ± standard deviation (M±SD).

To further clarify the substances with muscle enhancing effect in pea peptide, we divided soybean peptides into five components by HPLC (Figure 5A) and then detected the effects of each component on the activation of growth-signaling pathway in C2C12 cells. The results showed that all of five components obviously inhibited myostatin expression (*p* < 0.05); whereas the 3th, 4th, and 5th components significantly improved the phosphorylation of AMPK, and the 4th and 5th components tremendously elevated the expression of IGF-1R (Figures 5B–E). On the whole, the 5th component is likely superior to other components on the activation of growth-signaling pathway.

### Pea Oligopeptide 2 Largely Promoted the Proliferation of C2C12 Cells

**Figure 6:**
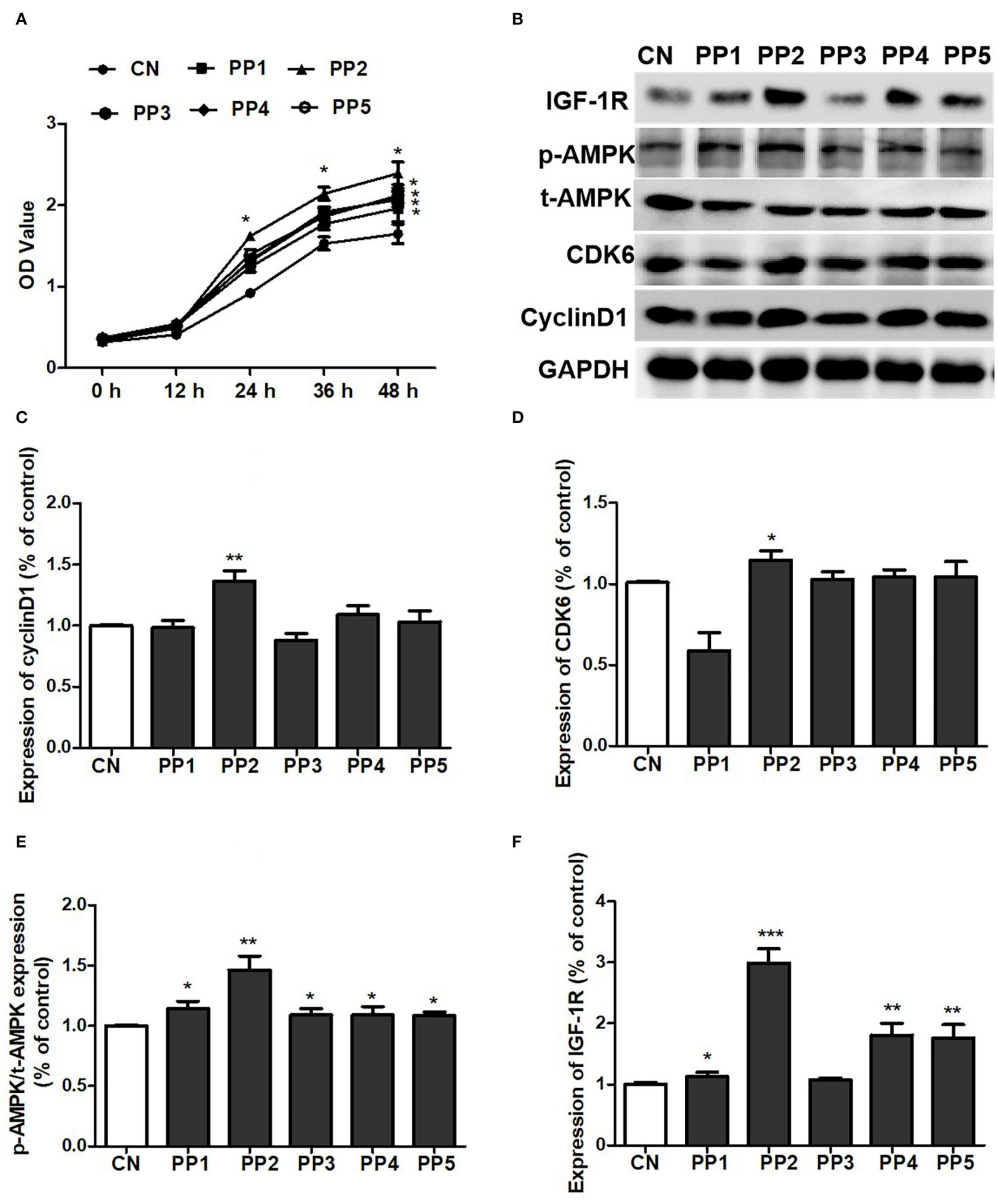
PP2 largely promoted the proliferation of C2C12 cells. **(A)** Effect of PP1, PP2, PP3, PP4, and PP5 on the growth of C2C12 cells; **(B)** effect of PP1, PP2, PP3, PP4, and PP5 on the expression of IGF-1R, p-AMPK, t-AMPK, cyclin D1, and CDK6; **(C)** statistical analysis of cyclin D1 expression; **(D)** statistical analysis of CDK6 expression; **(E)** statistical analysis of p-AMPK/t-AMPK expression; **(F)** statistical analysis of IGF-1R expression. The significant difference between groups was validated with one-way ANOVA using GraphPad Prism 6, *p < 0.05, **p < 0.01, ***p < 0.001 vs. CN group (n = 5/group). Data are presented as mean ± standard deviation (M ± SD).

We subsequently determined the amino acid sequence of the peptides in component 5 of pea peptides by mass spectrometry and found that the 5th component contained five oligopeptides (Table 2). Then, we synthesized these five oligopeptides and co-incubated with C2C12 cells. After treatment for 48 h, the five pea oligopeptides all significantly promoted the proliferation of C2C12 cells when compared to control (Figures 6B–F). Notably, co-incubation of PP2 resulted in a more conspicuous proliferation of C2C12 cells in comparison with other oligopeptides and presented an obvious cell growth promoting effect even at 24 h (Figure 6A). Correspondingly, western blot analysis also showed that co-culture PP2 significantly elevated the expression of IGF-1R, cyclin D1, CDK6, and p-AMPK when compared to control.

**Table 2:** Amino acid sequence of oligopeptide in pea peptide.

**Oligopeptide in pea peptide**	**Amino acid sequence**
1 (PP1)	Glu-Gly-Ser-Leu-Leu-Leu-Pro-His (EGSLLLPH)
2 (PP2)	Leu-Asp-Leu-Pro-Val-Leu (LDLPVL)
3 (PP3)	Leu-Leu-Tyr-Val-Ile-Arg (LLYVIR)
4 (PP4)	Thr-Asn-Tyr-Glu-Glu-Ile-Glu-Lys-Val-Leu-Leu (TNYEEIEKVLL)
5 (PP5)	Asn-Thr-Asn-Tyr-Glu-Glu-Ile-Glu-Lys-Val-Leu (NTNYEEIEKVL)

## Discussion

Nowadays, much evidence has confirmed that plant protein supplementation produces similar gains in muscle thickness and strength as animal proteins in response to resistance exercise (4, 13–15). In this study, we observed that compared to placebo, pea peptide supplementation combined with resistance exercise markedly increased the muscle thickness of biceps brachii and the muscle strength of upper limb. Meanwhile, we found that pea peptide combined with resistance exercise significantly increased the cross-sectional area of muscle fibers. These above results demonstrated that pea peptide plays a crucial role in the promotion of muscle growth in response to resistance exercise training. Skeletal muscle is broadly characterized by the presence of two distinct categories of muscle fibers named slow-twitch fibers (type I) and fast-twitch fiber (type II). Fast-twitch fibers have higher LDH activity and display a stronger contractility in contrast with slow-twitch fibers (4). In our study, immunofluorescence staining as well as LDH detection revealed that pea peptide supplementation combined with resistance exercise obviously enhanced the ration of fast muscle fiber.

In addition, we investigated the effect of pea peptide on muscle growth-signaling pathway. The results indicated that pea peptide supplementation combined with resistance exercise largely increased the expression of IGF-1R and the ration of p-AMPK/t-AMPK and conversely decreased myostatin expression. We also found that pea peptide supplementation combined with resistance exercise significantly elevated the concentration of circulating testosterone and IGF-1. IGF-1/IGF-1R signal axis plays a key role on muscle growth. It is reported that resistance exercise training effectively alleviated skeletal muscle atrophy by activating IGF-1/IGF-1R-PI3K/Akt pathway and resulted in the decrease of protein degradation and cell apoptosis (16). Myostatin, a member of the transforming growth factor-β superfamily, is a potent negative regulator of skeletal muscle growth and is conserved in many species, from rodents to humans. Myostatin is also known as growth differentiation factor 8. Myostatin inactivation can induce skeletal muscle hypertrophy, whereas its overexpression or systemic administration causes muscle atrophy (17). AMPK has also been considered to play an important role in regulating muscle mass and regeneration due to their effects on anabolic and catabolic cellular processes (18, 19).

To further explore the effect of pea peptide on the promotion of muscle growth, we obtained 5 components from pea peptides by HPLC and then incubated them with C2C12 cells to investigate the effect of various components on cell growth-signaling pathway. We observed that co-cultured with the 5th component of pea peptide significantly elevated the expression of p-AMPK and IGF-1R, while decreased myostatin expression. Subsequently, we harvested peak solution of the 5th component to determine the amino acid sequence using mass spectrometer. Additionally then, these five oligopeptides were synthesized and incubated with C2C12 cells to evaluate the effect of these five oligopeptides on cell proliferation. We found that all these five oligopeptides induced a significant proliferation of C2C12 cells after incubated for 48 h; moreover, PP2 incubation likely resulted in a more obvious increase in the number of cells than other oligopeptides. Further study indicated that PP2 induced the more conscious elevation on the expression of p-AMPK, IGF-1R, cyclin D1, and CDK6. Muscle regeneration is a rapid and extensive self-renewal process relying on the presence of satellite cells. These cells are activated and then proliferated to provide a myoblast population that either fuses to each other to create new myofibers or fuses to existing damaged myofibers for repair (20). According to a latest cell cycle study, an excessively activated CDK/cyclin D1 would cause the growth and development of cells. As a cell cycle regulator, cyclin D1 is necessary for the G1 phase progress (21). In the G1 phase, retinoblastoma is hyperphosphorylated by the cyclin DCDK4/CDK6 complex, causing the release and activation of the E2F-DP transcription factor complex, which in turn activates the expression of the genes required for S-phase progression (22). People have always been concerned about whether plant protein supplement can promote muscle growth as animal protein supplement in response to resistance exercise. Similar to other findings, our research provides strong evidence that pea protein can significantly promote muscle growth. In addition, this study also determines an optimal oligopeptide promoting muscle growth from pea protein. Of course, our results cannot resolve the debate between proteins and oligopeptides on the promotion of muscle growth.

In the recent years, muscle atrophy is a public health issue that causes a growing concern around the world, which imposes a huge burden on life quality of patients (23). Skeletal muscle is the largest organ in mammals and plays the vital roles in mobility and metabolism. Excessive loss of muscle mass is associated with poor prognosis in several diseases, including myopathies and muscular dystrophies, as well as in systemic disorders such as cancer, diabetes, sepsis, and heart failure (24), and thus, maintenance of muscle mass is crucial for preventing metabolic disorders. It is reported that an inadequate diet and especially a low diet in protein appear to be associated with a decrease in muscle mass and muscle strength (25, 26). To alleviate muscle atrophy, several researchers have even suggested that elderly individuals should consume more protein than recommended daily allowance (27–29). Resistance training is widely recognized to increase muscle mass and muscle tension strength. Therefore, it is now recommended to incorporate resistance training programs and an adequate protein intake for the maintenance of muscle mass and muscle strength (30, 31). In our study, we demonstrated that pea peptide supplementation combined with resistance exercise significantly promoted muscle growth. Based on other researches (32) and our results, we regarded that pea peptide supplementation combined with resistance exercise may be a good strategy for the prevention and treatment of muscle atrophy.

Taken together, our study demonstrated that pea peptide combined with resistance exercise training markedly promoted skeletal muscle growth. Furthermore, the oligopeptide with amino acid sequence of LDLPVL presented a more significant proliferation of C2C12 cells than other oligopeptides.

## Data Availability Statement

The raw data supporting the conclusions of this article will be made available by the authors, without undue reservation.

## Ethics Statement

The animal study was reviewed and approved by Animal Care and Use Committee at Wuhan Sports University.

## Author Contributions

WW and QW participated in the peptide purification and Western blots analysis. JK participated in HE and ELISA analysis. All authors contributed to the article and approved the submitted version.

## Conflict of Interest

WW, ZZ, XiZ, XuZ, and JL were employed by Zhong Shi Du Qing (Shandong) Biotechnology Company. QW, JK, and JD were employed by Amway (Shanghai) Innovation & Science Co., Ltd. The remaining authors declare that the research was conducted in the absence of any commercial or financial relationships that could be construed as a potential conflict of interest.

## Publisher's Note

All claims expressed in this article are solely those of the authors and do not necessarily represent those of their affiliated organizations, or those of the publisher, the editors and the reviewers. Any product that may be evaluated in this article, or claim that may be made by its manufacturer, is not guaranteed or endorsed by the publisher.
